# One- versus Two-Minute Intercostal Nerve Cryoanalgesia in Children Undergoing Surgery for Funnel Chest Deformity

**DOI:** 10.3390/jpm14080875

**Published:** 2024-08-18

**Authors:** Sławomir Zacha, Manuel Lopez, Jarosław Bilas, Karolina Skonieczna-Żydecka, Jakub Miegoń, Jowita Biernawska

**Affiliations:** 1 Department of Paediatric Orthopaedics and Musculoskeletal Oncology, Pomeranian Medical University, 70-252 Szczecin, Poland; 2 Pediatric Surgery Department, Vall d’Hebron Hospital, 08035 Barcelona, Spain; manuel.lopezparedes@vallhebron.cat; 3 Department of Biochemical Sciences, Pomeranian Medical University, 70-252 Szczecin, Poland; karolina.skonieczna.zydecka@pum.edu.pl; 4 Department of Anaesthesiology and Intensive Therapy, Pomeranian Medical University, 70-252 Szczecin, Polandjowita.biernawska@pum.edu.pl (J.B.)

**Keywords:** acute pain, cryoanalgesia, Nuss surgery, funnel chest

## Abstract

An inherent defect of the sternum and ribs results in the formation of a funnel-shaped anterior chest wall. The gold standard of surgical correction is the minimally invasive Nuss procedure, which might cause severe pain and carries the risk of sensory disturbances and chronic discomfort. Integrating cryoanalgesia with standard multimodal analgesia improves the outcomes of this procedure. Based on histological results, it was hypothesised that the time of cryo-application can be reduced from the current standard period of two minutes. The goal of this study was to evaluate the efficacy of a one-minute application compared with the routine two-minute method in the same patient, considering the subjective perception of pain and sensory disturbances. A total of 33 patients were included in this prospective study. The results show that the assessment of pain severity and sensory disturbances did not differ significantly in terms of the time of cryo-application during first 14 days after the surgical procedure. The one-minute cryo-application time for intraoperative intercostal nerve cryoablation prior to the Nuss procedure seems to be as safe and effective as the routinely used two-minute application time in regards to pain severity, sensory disturbances, and the risk of chronic pain development. Intercostal nerve cryoanalgesia is an essential element of multimodal analgesia.

## 1. Introduction

The most prevalent congenital defect of the sternum and adjacent ribs results in the formation of a funnel-shaped anterior chest wall [1]. Apart from aesthetic implications, this structural anomaly not only causes a significant decrease in the self-esteem of the affected individuals but also has a deleterious effect on their cardiopulmonary functionality [1,2].

The standard value of the Haller index is 2.5, and surgical deformity correction is required when the index exceeds the threshold of 3.2 [2].

Currently, the most pervasive approach is the minimally invasive Nuss surgical procedure [2]. Since its introduction in the late 1990s, the Nuss procedure has become the gold standard for pectus excavatum surgical correction.

Due to the inefficient effects of multimodal analgesia in the field of pectus surgery, additional pain management solutions are needed [3,4,5,6].

The use of low temperatures for pain relief dates back to the time of Hippocrates [7]. The modern notion of ‘nerve freezing’ is based on the Joule–Thomson phenomenon [8]. Histological studies show that freezing induces axonal damage while preserving the integrity of the perineurium and epineurium [7,9]. 

The first report on the use of cryoanalgesia during the Nuss procedure was published in 2016 [10]. The beneficial effects of cryoablation include reduced demand for strong analgesic medications, thereby reducing the associated side effects. Cryo-application allows for a faster return to independence and more effective post-operative rehabilitation, resulting in a shorter hospital stay and a reduction in the total costs of hospitalisation. 

The precise timing of the clinical analgesic effect of intercostal nerve cryoanalgesia remains unclear, with studies suggesting that the effect is not immediate [11]. In routine practice, freezing for two minutes per intercostal nerve is employed. However, histological studies have shown equivalent damage to nerves in terms of axonal degeneration using either one- or two-minute cryo-applications [9]. Several clinical results show that one-minute cryo-application in thoracotomy patients is effective [12,13]. Based on this knowledge, Zeineddin et al. conducted a study evaluating the efficacy of integrating multimodal analgesia with a one-minute application of nerve freezing compared with the results for a paravertebral block [14]. The researchers confirmed the many advantages of intraoperative cryoanalgesia, despite the shorter cryo-application time. However, the protocol of their study did not precisely define the assessment of sensory disturbances and pain type or its severity. To date, the present study is the only one that has assessed the efficacy of cryo-application for one minute per intercostal nerve during the Nuss procedure for funnel chest correction. 

Based on the consideration that pain is a subjective sensation, it is worth assessing its intensity in the individual patient. The same patient is subjected to both procedures simultaneously, one on each side of the chest, and their sensations on both sides are compared. To date, no other study has been designed in this manner.

Due to the positive preliminary histological and clinical results, the present study was designed to evaluate the efficacy of the one-minute application compared with the routine two-minute method in the same patient.

## 2. Materials and Methods

### 2.1. Study Design and Patients

This was a prospective, non-randomised pilot study. The perioperative management protocol was similar to that used in our two previous studies [15,16]. We included 33 patients under 18 years of age diagnosed with funnel-shaped anterior chest wall deformity undergoing minimally invasive Nuss surgery. The exclusion criteria were as follows:

- Thoracic deformity other than funnel chest;
- Advanced chronic respiratory or circulatory failure;
- Emergency surgery or reoperation;
- History of thoracotomy or thoracic surgery;
- Mental impairment precluding communication with the patient or lack of consent for cryoanalgesia or regional analgesia;
- History of allergy to local anaesthetic;
- History of taking medication for chronic pain [15].

The legal guardians of all study patients (and the patients themselves, if they were >16 years of age) provided their written informed consent to participate in the study. Our protocol was registered in the clinicaltrials.gov registry under NCT number 05831137. Approval was obtained from the Bioethics Committee of Medical University of Pomerania, Szczecin, Poland, under number KB-006/43/2022.

The study group consisted of 33 patients (mean age of 14 years and range of 11–18 years), 27 of whom were boys. All patients received regional anaesthesia (based on the erector spine plane block of the dorsal extensor muscle compartment, according to NYSORA guidelines) after the induction of general anaesthesia [17]. An intraoperative nerve block and multimodal analgesia were used, according to the same protocol as that employed in our previous studies [15,16].

### 2.2. Preparation for Surgery

The Haller index was determined based on chest computed tomography. A value greater than 3.2 was an indication for surgery. The admission plan was based on the principles of the enhanced recovery after surgery (ERAS) protocol. 

A preoperative questionnaire based on the modified Nuss questionnaire [18] was administered to the patient and parents. This is a two-step questionnaire for a paediatric patient (including both patient and parent questionnaires) that assesses the impact of the surgical procedure on the patient’s psychosocial and physical functioning. The questionnaires included 12 items for patients and 13 items for parents, with scores ranging from 1 to 4.

On the day of surgery, the patient received metamizole (15 mg/kg for children under 50 kg or 1 g orally for children over 50 kg), together with a carbohydrate-rich fluid (Preop; Nutricia, Poland), which was given orally 2 h before surgery [15].

### 2.3. Anaesthesia

The general concept of intraoperative pain management adopted was the same as that employed in our previous study [15]. All patients underwent general anaesthesia with double-lumen tube intubation, fibreoptic control of tube position, and mechanical ventilation using the Primus device (Drager). 

After the induction of general anaesthesia, an ESP block was performed in all patients using ultrasound (according to the NYSORA Nerve Blocks protocol, New York, NY, USA) and 0.25% bupivacaine at 0.3 mL/kg per side (not more than 20 mL per side) [15].

### 2.4. Surgical Procedure

The surgical procedure was previously described in our other articles [15,16,19]. Eligibility for surgery was based on a physical examination and a CT scan. All patients underwent surgery for funnel chest deformity using the modified Nuss procedure.

Intraoperative intercostal nerve cryoablation was performed using the Cryo-S Painless device. The A-30/300/PEA/R/RF probe, designed for intraoperative use, was inserted into the pleural cavity through the skin incision in the same intercostal space as the previously inserted plate. Adequate visualisation conditions were ensured through the continuous ventilation of one lung on the contralateral side. Cryoablation was routinely performed at six levels, usually Th4-Th10, depending on the deformity. The curved tip of the probe was placed in the extrapleural space (as this allows for better probe contact with the nerve) under the lower edge of the rib, approximately 2 cm laterally to the ends of the transverse processes. The duration of a single cryoablation application was 2 min per intercostal nerve on the right side and 1 min for each level on the left side. In total, the cryoablation procedure lasted approximately 25 min.

### 2.5. Postoperative Course

In addition to the assessment of the vital signs, a protocol was followed to assess pain intensity according to the NRS every 1 h for 24 h (when the patient was sleeping, the nurse marked 1 point) and then every 8 h until hospital discharge and to record the type, dose, and route of medication administered, as well as the occurrence of side effects and complications. In the postoperative period, the medications were administered at fixed intervals: paracetamol (15 mg/kg for children under 50 kg or 1 g for children over 50 kg) was orally or intravenously administered every 6 h; metamizole (15 mg/kg for children under 50 kg or 1 g for children over 50 kg) was orally or intravenously administered every 6 h; ibuprofen (10 mg/kg for children under 40 kg or 400 mg for children over 40 kg) was orally or intravenously administered every 8 h; and morphine infusion at 10–40 mcg/kg/h with an infusion pump and boluses at max 0.1 mg/kg were administered every 4 h, if the NRS score exceeded 3 points at rest or 6 points on exertion. Intravenous opioid infusion and transition to oral treatment were determined according to individual daily requirements. 

### 2.6. Definitions

The adverse effects of pharmacotherapy were defined, in accordance with our previous studies, as nausea, vomiting, dyspnoea, pruritus, constipation, urinary retention, dizziness, drowsiness preventing rehabilitation, apnoea, hypotension according to age-specific physiological values, bradycardia, and drop in oxygen saturation to <90% [15,16].

The complications of cryoablation were defined as neuropathic pain, hyposensitivity, tingling/numbness, and hypersensitivity of the operated area (touch as a stimulus was tested along the anterior and posterior chest wall dermatomes from Th2 to Th8).

The complications of Nuss surgery were defined as respiratory and circulatory failure, the pneumothorax requiring drainage, haematoma, surgical site infection, pleural effusion or abscess, pericarditis, plate dislocation requiring reoperation, pain not relieved by standard means, pneumonia, cardiac perforation, and death.

### 2.7. Outcomes

We assessed the following outcomes:

- Acute pain intensity (maximum) up to the first 24 h after surgery, based on the patients performing a self-assessment every 1 h for 24 h using the NRS numerical scale (0–10 points) and reporting to the nurse;
- Acute pain intensity (maximum) on the first day after surgery, based on the patients performing a self-assessment every 1 h for 24 h (then every day for 14 days) using the NRS numerical scale (0–10 points) and referring to the parent;
- Length of time in which there was a need for intravenous opioid administration (days after surgery);
- Quality of post-operative rehabilitation in terms of accuracy of exercises and achievement of physical activity independence (days after surgery), based on self-assessment;
- Duration of surgery from skin incision to skin suture (minutes);
- Length of stay (LOS) in hospital (days);
- Occurrence of adverse reactions to pharmacotherapy and anaesthesia;
- Occurrence of complications after cryoablation and Nuss surgery [15,16].

Patients were asked to rate and record the severity of pain (on the NRS), hypoesthesia, hyperesthesia, and numbness.

After hospital discharge, the first scheduled follow-up was 14 days after surgery. The effect of surgery, pain location and its intensity, chest wall paraesthesia, and the results of the DN4 neuropathic pain questionnaire were assessed, and the patients’ notes were analysed. 

Full activity, chest wall pain and paraesthesia, the modified Nuss questionnaires for patients and parents, and the DN4 questionnaire were assessed again after 3 months. 

**Figure 1:**
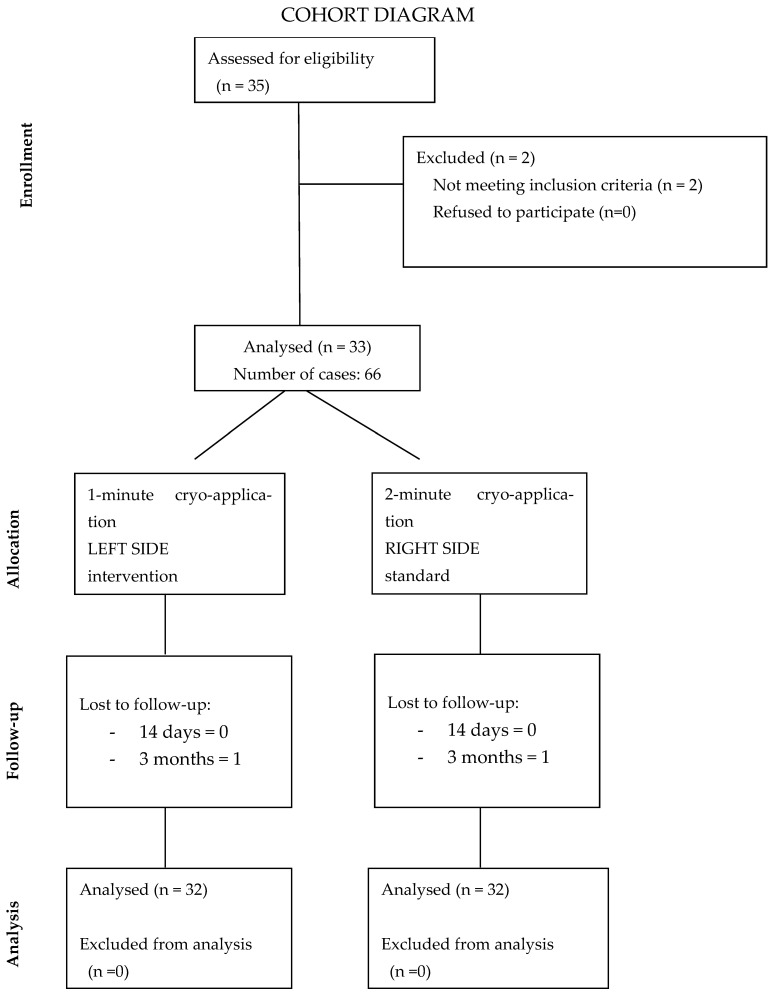
Flow diagram.

The flow diagram in Figure 1 shows the flow of patients throughout the study.

#### Statistical Analysis

The statistical analyses were performed using MedCalc statistical software, version 22.021 (Ostend, Belgium). A two-tailed *p* < 0.05 was considered to indicate a statistically significant difference. The Shapiro–Wilk test was used to test the normality of the distribution of continuous data. Consequently, these were expressed as medians ± interquartile ranges (IQRs). Categorical data were expressed as numbers (percentages). The paired Wilcoxon signed-rank test was used to compare the data. The McNemar test was used to assess the change in the frequency of sensory disturbances, and McNemar plots were created using Python’s data analysis and visualisation libraries, primarily Pandas and Matplotlib.

## 3. Results

### 3.1. Patient Characteristics

The study group consisted of 33 patients who underwent the modified Nuss operation for funnel chest correction. Boys represented 81% of the study group. One patient reported mild asthma, and no other comorbidities were reported. Demographic data, age, BMI, and Haller index scores are shown in Table 1. The American Society of Anesthesiology (ASA) scale score was 1 in 32 cases. 

All patients underwent a bilateral ESP block and intercostal nerve cryoanalgesia at six levels during thoracoscopy immediately before the Nuss procedure.

The analysis of the parameters in the perioperative period is shown in Table 2.

The ability to change body position independently and to perform exercises in any position (independence) was achieved by 1 patient on the first postoperative day and by 97% of patients (32 patients) on the second day.

### 3.2. Adverse Effects

The adverse effects after pharmacotherapy involved postoperative nausea and vomiting, which were observed in three cases. No features of local anaesthetic toxicity (LAST) or complications of the ESP blockade were observed.

Complications from the Nuss procedure were observed in three patients (plate dislocation requiring revision in two cases and mild pneumothorax in one case). 

### 3.3. Pain and Sensory Assessment

Pain assessment data are shown in Table 3.

**Figure 2:**
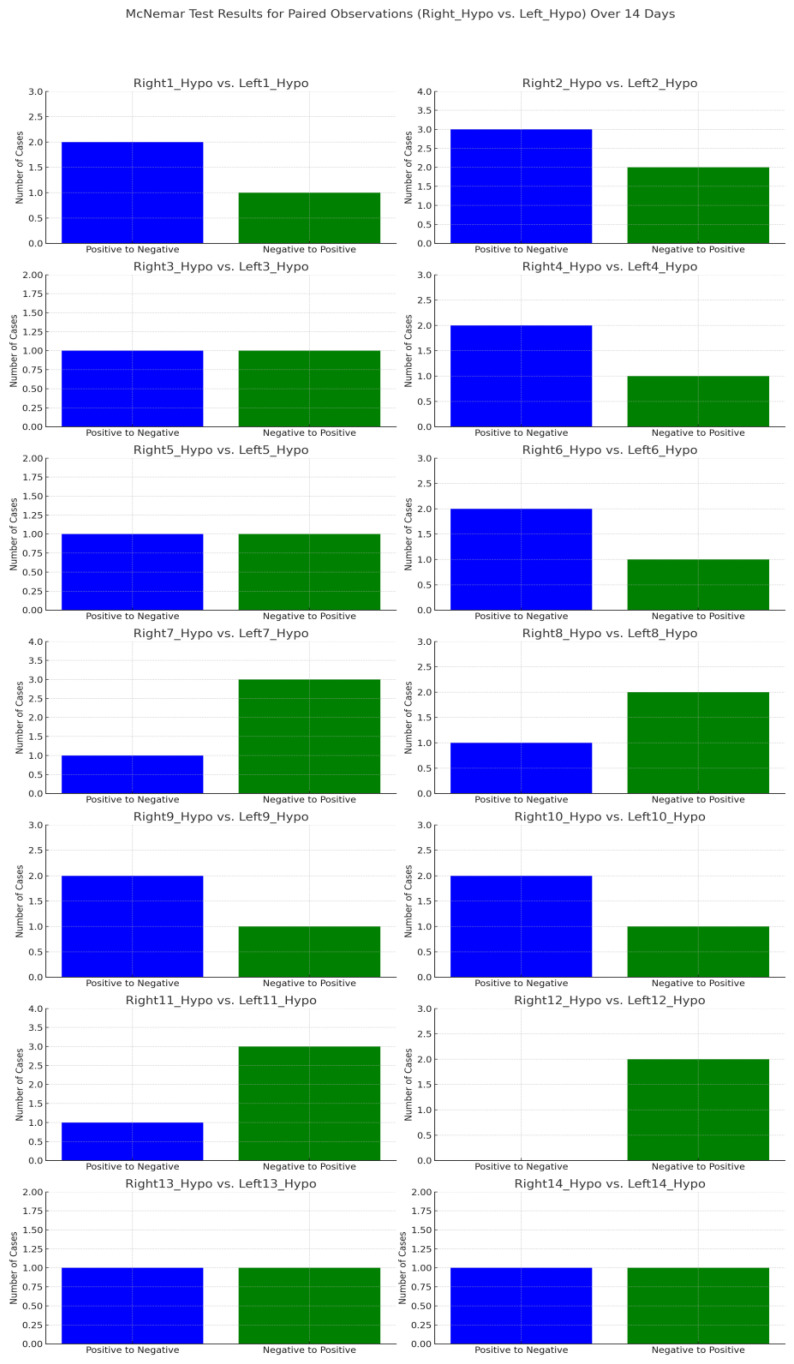
McNemar test results for paired observations: hyposensitivity.

The data of the sensory assessment are depicted in Table 4, Table 5 and Table 6. The exemplary representation of the McNemar test results for hyposensitivity is shown in Figure 2.

The DN4 score was 1 point (range of 1–2 IQR) 14 days after surgery for both the left and right sides. Three months after surgery, the range was 1 point (IQR of 0–1 points). None of the above results regarding pain severity and sensory disturbances differed significantly in terms of the time of cryo-application (one versus two minutes). There were no differences between either side of the chest in any of 16 cases of sensory dysfunction occurrence.

The 3-month follow-up data are presented in Table 7. 

The results of the modified Nuss questionnaire showed significant improvement in both the children’s and parents’ responses (<0.0001 and <0.0001, respectively, according to the Wilcoxon test of paired samples). 

## 4. Discussion

The results of our study demonstrate the comparison of one- versus two-minute cryoanalgesia of the intercostal nerves during minimally invasive Nuss surgery in patients diagnosed with funnel chest deformity and confirm the equal intensity of acute postoperative pain and need for intravenous opioid treatment following cryoanalgesia with different application times. This method also allowed for equivalent achievement of motor independence and adequate performance of exercises during postoperative rehabilitation. We also found no differences in sensory disturbances within the first 14 days after surgery.

The current International Association for the Study of Pain (IASP) definition of pain as, ‘an unpleasant sensory and emotional experience associated with, or described in terms of, actual or potential tissue damage’ was recommended by the Subcommittee on Taxonomy and was adopted by the IASP Council in 1979, with the caveat that ‘pain is always subjective’ [20]. This definition has been widely accepted by many health professionals and researchers, including the World Health Organisation. Recognising the subjectivity of pain perception, we designed a study in which the same patient was asked to rate the effectiveness of intercostal nerve cryoanalgesia application time, with a one-minute application period compared to the standard two-minute method. This is the first study evaluating the effectiveness of one-minute cryoanalgesia according to the intensity of postoperative pain and sensory disturbances in the same patient. We evaluated the efficacy of different periods of low-temperature application directly to the intercostal nerve region (extrapleural) during Nuss surgery. 

The successful effect of intraoperative intercostal nerve cryoanalgesia as an additional method of multimodal analgesia during Nuss surgery has been confirmed in clinical studies. The use of the standard freezing time of two minutes per nerve results in the reduction in the intensity of acute postoperative pain and a significantly lower pain rating scale score [21,22,23,24,25]. Clinical evaluation of the efficacy of a shorter low-temperature application time during Nuss surgery was only performed previously in one study, that of Zeineddin et al., who evaluated the effect of one-minute intercostal nerve freezing for postoperative analgesia after surgical correction of pectus excavatum [14]. They confirmed the previously documented observation that this treatment allows for a reduction in hospital length of stay and total treatment costs. The optimal time for low-temperature application is unknown, and the duration of the clinical effect depends on the time of application. If the freezing time is prolonged, nerve ablation may occur, causing permanent damage. Therefore, to achieve an analgesic effect while maintaining safety, in this study, the authors proposed the introduction of a protocol for a shorter application time, based on the results of histological studies [9,12,13]. However, compared with the protocols of previous research, we used a different type of cryo-probe and different sites for cryo-application, as the latter have been previously determined based on two-lung ventilation using a standard endotracheal tube; in our study, we used one-lung ventilation during the cryo-procedure to optimise direct visualisation. We used the Cryo-S Painless device, developed by the Polish company Metrum Cryoflex, in conjunction with the A-30/300/PEA/R/RF cryogenic probe, which is specifically designed (in a ‘hockey stick’ shape) for intraoperative cryoanalgesia during thoracoscopy [26]. In Poland, the first intraoperative cryoanalgesia of intercostal nerves during Nuss surgery in children was conducted in May 2022 at the Department of Paediatric Orthopaedics and Musculoskeletal Oncology of Pomeranian Medical University in Szczecin [15,16].

Regarding the subjective perception of pain intensity, we believe that the concept of comparing two groups of patients may affect the reliability of comparing the effectiveness of different cryoanalgesia treatment times. Therefore, we compared the effectiveness of the two freezing times between the left and right sides of the chest in the same patient. We assessed the maximum pain intensity during the first 24 h and for 14 consecutive days after the procedure. Simultaneously, we assessed whether sensory disturbances (hypoesthesia, hyperalgesia, and tingling/numbness) occurred during the same time intervals. Three months after surgery, we determined whether chronic pain had occurred. The results of our study prove that the cryo-application times of one and two minutes were equal in terms of the effects assessed, confirming the safety of the shorter application time. It should be emphasised that the design of our study concerned a procedure performed during thoracoscopy, where the extrapleural location of the probe tip and direct visualisation allowed for precise probe positioning. However, our results of the one-minute cryoablation procedure should not be directly extrapolated to a procedure performed percutaneously.

Zeineddin et al. also analysed the risk of neuropathic pain in a study evaluating the efficacy of one-minute cryoanalgesia. However, the authors did not report the criteria for the diagnosis of neuropathic pain according to specific scales. Their protocol included starting gabapentin therapy in patients who were confirmed to feel pain after 7 days of treatment with non-steroidal analgesics. The authors described the occurrence of neuropathic pain in both the regional analgesia group and the cryoanalgesia group, and the differences were not statistically significant. Studies evaluating the safety of cryoanalgesia compared to other analgesic strategies after Nuss surgery have not proved an increased incidence of neuropathic pain in either adults or children [23,26,27,28,29,30]. In 2008, a study was conducted to evaluate the efficacy of intercostal nerve freezing (90 s application at −70 °C) compared with the that using epidural catheter analgesia after thoracotomy in relation to the risk of neuropathic pain. There was a significantly higher incidence of allodynia-like pain in the cryoanalgesia group, but the mechanism of this phenomenon was not explained. In the study by Eldredge et al. evaluating the incidence of paraesthesia after Nuss surgery, neuropathic pain was observed in 13% of patients under 21 years of age [28]. None of the patients required pharmacological treatment. In subsequent studies describing the incidence of neuropathic pain in children after Nuss surgery, the prevalence rates ranged from 0 to 6% [23,29]. However, the methodology of the above studies did not define the diagnosis of neuropathic pain. Our study protocol described the diagnosis of neuropathic pain using a specific DN4 scale; we found no cases of neuropathic pain in our group.

In the available literature, there are no studies evaluating the severity of sensory dysfunction in relation to different cryo-application times. On the other hand, the occurrence of sensory disturbances in the form of hyposensitivity, hypersensitivity, or tingling after thoracic surgery has been described in several studies, regardless of the use of cryoanalgesia [23]. This is due to the technique of the procedure itself, i.e., the method of plate fixation used for correction that causes compression and damage to the intercostal nerves. Eldredge et al., in a prospective study, evaluated the effect of using two-minute cryoanalgesia of the intercostal nerves during Nuss surgery. The frequency and type of paraesthesia were analysed, and the results show that the occurrence of hypersensitivity was limited to two dermatomes in 46% of cases and involved more than three dermatomes in 10% of patients [28]. The results of other retrospective studies describing the occurrence of paraesthesia after 2 min of cryoanalgesia during Nuss surgery indicated prevalence rates of 0–23% [28,29]. In our study, during the first 2 weeks after surgery, hyposensitivity was reported by one–two patients, only on the right side (treated with two-minute cryo-application). Similarly, selective hyposensitivity occurred on the left side in one–two patients (treated with one-minute cryo-application). Irrespective of the duration of freezing, bilateral hyposensitivity was reported in approximately 30% of patients in the first 7 days after the procedure and in 21% of the children afterwards. These differences were not statistically significant. Finally, 3 months postoperatively, 31% of the children reported decreased tactile sensation of the anterior chest wall. Hypersensitivity of the anterior chest wall, depending on the duration of cryo-application in the first 14 days after surgery, was reported by 9–20% of patients, and the differences were not statistically significant. After 3 months, clinically insignificant hypersensitivity (no treatment required) was reported in 18% of patients, and there were no differences between either side of the chest in any of these cases of sensory dysfunction occurrence.

The risk of chronic pain after thoracic surgery is 30–50%, according to the literature [6]. In our study, the occurrence of pain at rest was observed in one patient and on exertion in six patients (18%) 3 months after surgery. In our opinion, this is a significant reduction of the chronic pain risk resulting from the cryoanalgesia procedure, irrespective of the duration of cryo-application. The use of preoperative regional anaesthesia (a bilateral ESP block, in our protocol), together with the current standard of multimodal analgesia, reduces the risk of chronic pain. Our observations coincide with Eldredge’s results [28].

Regarding the increased risk of chronic pain after cryoanalgesia during thoracotomy, as described by the authors in a 2008 study, it is important to consider whether or not this observation was due to failure to perform pain management optimisation in compliance with the principles of multimodal analgesia, including regional analgesia.

Our study has several limitations due to the small number of patients assessed. We also noticed that the NRS scores reported at home by the children were higher than those noted in hospital. We could not determine whether or not the patients’ answers depended upon the patient’s attitude towards the person asking the question.

## 5. Conclusions

The one-minute application time for intraoperative intercostal nerve cryoablation prior to the Nuss procedure seems to be as safe and effective as the routinely used two-minute application time in terms of resulting pain severity, sensory disturbances, and the risk of chronic pain development. Cryoanalgesia is an essential part of multimodal analgesia.

## Figures and Tables

**Table 1:** Demographic data and preoperative assessment of study population.

Parameter [Mean ± SD]	Study Group (*n* = 33)
Age (years)	14.6 ± 1.7
BMI	18 ± 1.6
Haller index	3.6 ± 0.45
Child survey before surgery (points)	28 ± 10
Parent survey before surgery	31 ± 6

Legend: *n*—number; BMI—body mass index.

**Table 2:** Parameters measured in perioperative period.

Parameter [Mean ± SD]	Study Group (*n* = 33)
Surgery time (minutes)	66.81 ± 14.7
Length of hospitalisation (days)	3.9 ± 0.5
Discontinuation of intravenous opioids after 24 h, *n* (%)	32 (97)
MAX NRS 24 h = 1, *n* (%)	2 (6)
MAX NRS 24 h = 2, *n* (%)	3 (9)
MAX NRS 24 h = 3, *n* (%)	28 (85)

Legend: *n*—number; MAX NRS 24 h—acute pain intensity value (maximum) according to the NRS scale during first 24 h, as reported by the patient to the nurse.

**Table 3:** The comparison of the pain severity in right and left sides of the chest reported to the parents.

Parameter	Right Side—Standard (Points) 2 min per Nerve	Left Side—Intervention (Points) 1 min per Nerve	*p*
MAX NRS 1 POD	6	6	0.1635
MAX NRS 2 POD	5	5	0.4054
MAX NRS 3 POD	5	5	0.9517
MAX NRS 4 POD	5	5	0.4101
MAX NRS 5 POD	5	4	0.3763
MAX NRS 6 POD	4	4	0.0571
MAX NRS 7 POD	4	4	0.0884
MAX NRS 8 POD	4	4	0.4364
MAX NRS 9 POD	3	3	0.0740
MAX NRS 10 POD	3	3	0.1635
MAX NRS 11 POD	3	3	0.0597
MAX NRS 12 POD	2	2	0.0759
MAX NRS 13 POD	2	2	0.4762
MAX NRS 14 POD	2	2	0.0532

Legend: *n*—number, according to the Wilcoxon test (paired samples); POD—postoperative day; MAX NRS—acute pain intensity value (maximum), according to the NRS scale (medians).

**Table 4:** The comparison of the sensory disturbance hyposensitivity reported on the right and left sides of the chest (self-assessment of the patient, reported to the parent).

Hyposensitivity	Right Side Only (*n*)	Left Side Only (*n*)	*p*	Both Sides (*n*, %)
1 POD	1	2	1	13 (39)
2 POD	2	3	1	15 (45)
3 POD	1	1	1	13 (39)
4 POD	1	2	1	12 (36)
5 POD	1	1	1	14 (42)
6 POD	1	2	1	10 (30)
7 POD	3	1	0.62	8 (24)
8 POD	2	1	1	7 (21)
9 POD	1	2	1	5 (15)
10 POD	1	2	1	6 (18)
11 POD	3	1	0.62	6 (18)
12 POD	2	0	0.5	6 (18)
13 POD	1	1	1	6 (18)
14 POD	1	1	1	6 (18)

Legend: POD—postoperative day.

**Table 5:** The comparison of the sensory disturbance hypersensitivity reported on the right and left sides of the chest (self-assessment of the patient, reported to the parent).

Hypersensitivity	Right Side Only (*n*)	Left Side Only (*n*)	*p*	Both Sides (*n*, %)
1 POD	1	1	1	6 (18)
2 POD	2	1	1	3 (9)
3 POD	1	2	1	4 (12)
4 POD	2	1	1	4 (12)
5 POD	0	1	1	5 (15)
6 POD	1	2	1	7 (21)
7 POD	1	3	0.62	8 (24)
8 POD	3	1	0.6	10 (30)
9 POD	3	2	1	9 (28)
10 POD	3	3	1	7 (21)
11 POD	2	2	1	5 (15)
12 POD	3	2	1	4 (12)
13 POD	1	0	1	6 (18)
14 POD	1	1	1	5 (15)

Legend: POD—postoperative day.

**Table 6:** The comparison of the sensory disturbance numbness of the chest (self-assessment of the patient, reported to the parent).

Numbness	Right Side Only (*n*)	Left Side Only (*n*)	*p*	Both Sides (*n*, %)
1 POD	0	0	-	4 (12)
2 POD	1	1	1	4 (12)
3 POD	2	1	1	4 (12)
4 POD	1	0	1	4 (12)
5 POD	3	0	0.25	2 (6)
6 POD	4	0	0.12	1 (3)
7 POD	2	0	0.5	2 (6)
8 POD	1	0	1	3 (9)
9 POD	2	0	0.5	3 (9)
10 POD	1	0	1	1 (3)
11 POD	0	0	-	1 (3)
12 POD	0	1	1	2 (6)
13 POD	0	0	-	1 (3)
14 POD	0	0	-	1 (3)

Legend: POD—postoperative day; -—cannot perform test.

**Table 7:** Data at 3-month follow-up.

Parameter (*n*, %)	Study Group (*n* = 32)
Full activity	32 (100)
Pain at rest	1 (3)
Pain during activity	6 (18)
Hyposensitivity	10 (31)
Hypersensitivity	6 (18)
Neuropathic pain DN4	0
Child survey (after–before surgery) *	18 (47–27)
Parent survey (after–before surgery) *	19 (50–30)

Legend: DN4—neuropathic pain questionnaire; *—points, median value.

## Data Availability

The raw data are available upon request from the corresponding author.
